# Defining the malaria burden in Nchelenge District, northern Zambia using the World Health Organization malaria indicators survey

**DOI:** 10.1186/1475-2875-13-220

**Published:** 2014-06-05

**Authors:** Michael Nambozi, Phidelis Malunga, Modest Mulenga, Jean-Pierre Van Geertruyden, Umberto D’Alessandro

**Affiliations:** 1 Department of Clinical Sciences, Tropical Diseases Research Center, P.O Box 71769, Ndola, Zambia; 2 Unit international health, University of Antwerp, Antwerp, Belgium; 3 Medical Research Council Unit, Serrekunda, the Gambia; 4 Institute of Tropical Medicine, Antwerp, Belgium

**Keywords:** Malaria indicator survey, Zambia, Children, Endemicity

## Abstract

**Background:**

Malaria is considered as one of the major public health problems and among the diseases of poverty. In areas of stable and relatively high transmission, pregnant women and their newborn babies are among the higher risk groups. A multicentre trial on the safety and efficacy of several formulations of artemisinin-based combination therapy (ACT) during pregnancy is currently on-going in four African countries, including Zambia, whose study site is in Nchelenge district. As the study outcomes may be influenced by the local malaria endemicity, this needs to be characterized. A cross-sectional survey to determine the prevalence and intensity of infection among <10 years old was carried out in March-April 2012 in Nchelenge district.

**Methods:**

The sampling unit was the household where all children < 10 years of age were included in the survey using simple random household selection on a GPS coded list. A blood sample for determining haemoglobin concentration and identifying malaria infection was collected from each recruited child.

**Results:**

Six hundred thirty households were selected and 782 children tested for malaria and anaemia. Prevalence of malaria infection was 30.2% (236/782), the large majority (97.9%, 231/236) being *Plasmodium falciparum* and the remaining ones (2.1%, 5/236) *Plasmodium malariae*. Anaemia, defined as haemoglobin concentration <11 g/dl, was detected in 51.2% (398/782) children.

**Conclusion:**

In Zambia, despite the reported decline in malaria burden, pockets of high malaria endemicity, such as Nchelenge district, still remain. This is a border area and significant progress can be achieved only by concerted efforts aimed at increasing coverage of current control interventions across the border.

## Background

Malaria remains a major public health problem and a disease of poverty. In areas of stable and relatively high transmission, pregnant women and their newborn babies bear a high burden of malaria morbidity and mortality [1].

In Zambia, sulphadoxine-pyrimethamine (SP) is used for intermittent preventive treatment in pregnancy (SP-IPTp) during the second and third trimester. Until recently, malaria in pregnancy was treated with quinine, which has now been replaced by artemether-lumefantrine (AL) for women in the 2nd and 3rd trimester in pregnancy; quinine is used for the management of malaria during the 1st trimester and for severe cases [2,3]. To provide additional evidence on safety and efficacy of artemisinin-based combination therapy (ACT) during pregnancy, a multicentre trial (PREGACT, Clinical Trials identifier NCT00852423) is currently on-going in four sub-Saharan African countries, including Zambia.

The Zambian study site is located in Nchelenge district, Luapula province, at the border with the Democratic Republic of Congo (DRC). This is an area of intense malaria transmission as the prevalence of malaria infection in children aged <5 years was 21.8% in 2008 (national average 10.3%) and that of anaemia (Haemoglobin [Hb] <11 g/dl) 55.9% (national average 49.0%) [4]. In 2010, the prevalence in under 5 years was as high as 50.5% in some age groups and anaemia at 79.7% [5]. The same malaria indicator survey provided information on the coverage of key malaria interventions, i.e. prompt effective case management, insecticide-treated mosquito nets (ITNs), indoor residual spraying (IRS), and IPTp [6]. Considering that some of the end points of the trial on ACT in pregnancy, e.g. incidence of recurrent infections, can be influenced by the local malaria endemicity [1] and that no new information had been collected since 2008, a malariometric survey was carried out in Nchelenge district in March-April 2012 [7,8].

The rationale of this study was that it gives a more recent data on the prevalence of malaria in a high endemic region. In places like Zambia, where overall figures indicate that malaria is declining, there are still pockets of high endemicity, which are complicated by perfect breeding grounds for anopheles mosquito and cross-border human movements. This could affect national and global malaria control strategies. The primary objective was to estimate the prevalence and risk factors for malaria in Nchelenge, Luapula Province, Zambia.

## Methods

Nchelenge district, Luapula province, is located on the swampy shores of Lake Mweru, and has a population of 178,000 inhabitants, mostly peasant farmers and/or fishermen. The district has one first level hospital, ten rural health centres and two health posts. The survey was done in the catchment areas (total population of 43,105) of the two health centres, Kashikishi and Nchelenge, where the PREGACT trial is currently implemented.

The household was taken as the sampling unit. The households in Nchelenge and Kashikishi health centre catchment areas were previously numbered using the Global Positioning System (GPS). Then the households were selected according to a pre-defined, computer-generated list of random numbers [9]. In selected households, all children below the age of 10 years were included in the survey after having obtained the signed informed consent from the parent/guardian. Two questionnaires were administered, the household questionnaire and the parent/guardian’s questionnaire which were adapted from those recommended for the malaria indicators survey [10,11]. The household questionnaires include information on basic demographic and socioeconomic characteristics of the households.

The sample size was determined by assuming that malaria prevalence was 22% [4]; using Roasoft sample size calculator [12], 261 sampling units or households were needed to estimate such prevalence at 5% precision with a 95% confidence interval. Nevertheless, because of possible clustering within households, was assumed a conservative design effect of 2 was assumed, and a 20% adjustment was made for non-response (from household refusals or abandoned households). Based on these assumptions, it was estimated that 630 households would provide an estimate of prevalence with the desired precision.

All children <10 years of age in the selected households were asked to be included in the survey. Each study, subject was assigned a unique identifier for identification of biological samples and questionnaire data and to guarantee confidentiality. Information on bed net use, which was by observation, including long-lasting insecticidal nets (LLIN) or previous indoor residual spraying (IRS) was collected onto the structured questionnaires. A blood sample for a malaria rapid diagnostic test (RDT), (SD BIOLINE, Malaria Ag P.f., Standard Diagnostic Inc, Korea), thick blood film and for measuring haemoglobin (Hb) (Hemocue 301 machine, Angelholm, Sweden) was collected by finger prick. Children with fever (axillary temperature ≥37.5°C) and a positive RDT or only a positive RDT were treated with AL, the first-line treatment in Zambia. Children found to be sick or anaemic were referred to the health care centres for further management.

Thick blood films were stained with 10% Giemsa for 10 minutes. Two hundred high power fields were read before declaring the slide negative. The parasite density was determined by counting the number of parasites against 200 white blood cells (WBC) and assuming 8,000 WBC per μl. All stained slides were read by two independent microscopists unaware of the RDT results. In case of discrepant results between the two independent microscopists, a third microscopist read the slide until reaching a satisfactory agreement on positivity/negativity or the parasite density.

Data were double entered, cleaned and analysed using Epi Info™ (Center for Disease Control and Prevention, version 7.1.1.14). Further multivariable logistic regression analyses were performed using SAS version 9.2 (SAS Institute Inc., Cary, NC, USA). Mean and standard deviation were computed to describe the distribution of continuous variables, while the distribution of categorical variables was described using counts and proportions. Geometric mean and median were computed for continuous variables that did not follow a normal distribution: parasite densities and Hb concentrations. Continuous variables were compared between age groups using t-tests and categorical variable proportions by χ^2^ test or Fisher’s exact test. To account for uncertainty in the prevalence estimates, the 95% confidence intervals were computed. Anaemia was defined as Hb < 11 g/dl and created a dichotomous variable where Hb ≥ 11 g/dl was defined as non-anaemic. The risk of anaemia and malaria was estimated by fitting a multivariable logistic regression model and adjusted for possible risk factors to compute odds ratios (OR) and 95% confidence intervals (95% CIs). The variables included in the multivariable logistic regression model were selected a priori via extensive search of peer-reviewed literature. A two-sided p-value of ≤0.05 was used as threshold for statistical significance. The ‘survey logistic’ procedure in SAS 9.2 (SAS Institute Inc., Cary, NC, USA) with the option ‘clustering’ to account for the design effect of clustering within households.

Before the survey, the study protocol was approved by the scientific and technical committee (STC) of Tropical Diseases Research Centre (TDRC), the TDRC Ethical committee and the Ministry of Health. Participation in the survey was voluntary and participants were free to withdraw any time during the capture.

## Results

669 households were screened, 39 households were not responsive (21 were not available while 18 refused to participate). There was a 630/669 (94.2%) response. At this rate, the results presented are less biased. Out of the households that were responsive majority reported tubed well or borehole as main source of drinking water (87.5, 95% CI, 84.6-89.9). 90.0% (95% CI, 87.3-92.2) households had open pits or pit latrines without slabs as toilet facilities. 96.0% (95% CI, 94.1-97.4) households reported charcoal as the main source of household fuel. Most houses were made of finished brick walls (84.8%, 95% CI, 81.7-87.4) and grass thatched roofs or sticks and mud roofs (94.6%, 95% CI). In terms of owning durable goods, 83.5% (95% CI, 79.7-87.6) households owned a landline telephone or a cell phone, while a majority (64.3%, 95% CI, 60.4-68.0) also owned a bicycle (Table 1).

Seven hundred and eighty two children distributed in 630 households were enrolled in the study; girls (59.5%, 465/782) were more represented than boys (40.5%, 317/782) (p < 0.005). The median age was five years (IQR, 3–8), with the 7–10 years age group as the largest age group (37.9%, 296/782). Only about a third of children sampled (35.9%, 281/782, 95% CI, 32.6-39.4) came from a household with a bed net. Among those coming from a household with bed net 78.3% (220/281, 95% CI, 73.5-83.1) reported to have used it the previous night. Coverage of IRS was even lower, only 11.1% (87/782, 95% CI, 9.1-13.6) households had been sprayed by the local district health team (Table 1).

**Table 1:** Baseline and selected socio-economic characteristics in children from Nchelenge District, Zambia (95% CI)

**Variable**	***%***
Males (n/N)	40.5 (317/782)
Median age in years (IQR)	5 (3-8)
Bed net ownership (95% CI)	35.9 (32.6-39.4)
Slept under a bed net last night (95% CI)	78.3 (73.5-83.1)
Taken anti-malarial treatment during last month (95% CI)	63.7 (60.2-67.0)
Indoor residual spraying last year (95% CI)	11.1 (9.1-13.6)
Mother’s education (95% CI)	
Primary	77.0 (73-82)
Secondary	20.0 (16-25)
Tertiary	2.0 (1-4)
Average number of individuals/household (95% CI)	3.2 (3.1-3.3)
Source of drinking water (95% CI)	
Pipe into dwelling/yard/plot/public tap	1.4 (0.4-4.5)
Tube well/borehole	87.5 (84.6-89.9)
Dug well	6.5 (4.3-10.0)
Surface water: river/dam/lake/spring/pond	4.1 (2.0-8.2)
Toilet type (95% CI)	
Flush or pour flush	2.6 (1.1-6.0)
Ventilated improved pit latrine/pit latrine with slab	6.0 (3.8-9.5)
Pit latrine without slab/open pit	90.0 (87.3-92.2)
Compositing toilet/hanging toilet latrine/toilet	0.8 (0.1-3.6)
House wall material (95% CI)	
Natural wall	5.7 (4.0-8.7)
Rudimentary wall	4.4 (2.6-8.3)
Finished wall bricks	84.8 (81.7-87.4)
Finished wall cement/stone with lime/cement blocks	4.6 (2.4-8.7)
Roofing material (95% CI)	
Natural roof thatch/sticks and mud	94.6 (91.7-97.61)
Rudimentary roof	0.3 (0.1-1.27)
Finished roof iron/wood/calamine/concrete/shingles	4.6 (2.8-8.4)
Source of fuel (95% CI)	
Electricity/biogas/kerosene/coal/lignite	1.0 (0.1-4.6)
Charcoal	96.0 (94.1-97.4)
Firewood/straw	1.4 (0.7-2.8)
Household durable goods (95% CI)	
Radio	22.2 (19.1-25.7)
Television	3.3 (2.1-5.1)
Mobile or landline phone	83.5 (79.7-87.6)
Refrigerator	1.8 (0.9-3.2)
Bicycle	64.3 (60.4-68.0)
Motor cycle/scooter	0.48 (0.1-1.5)
Car/truck	1.1 (0.5-2.4)

**Figure 1:**
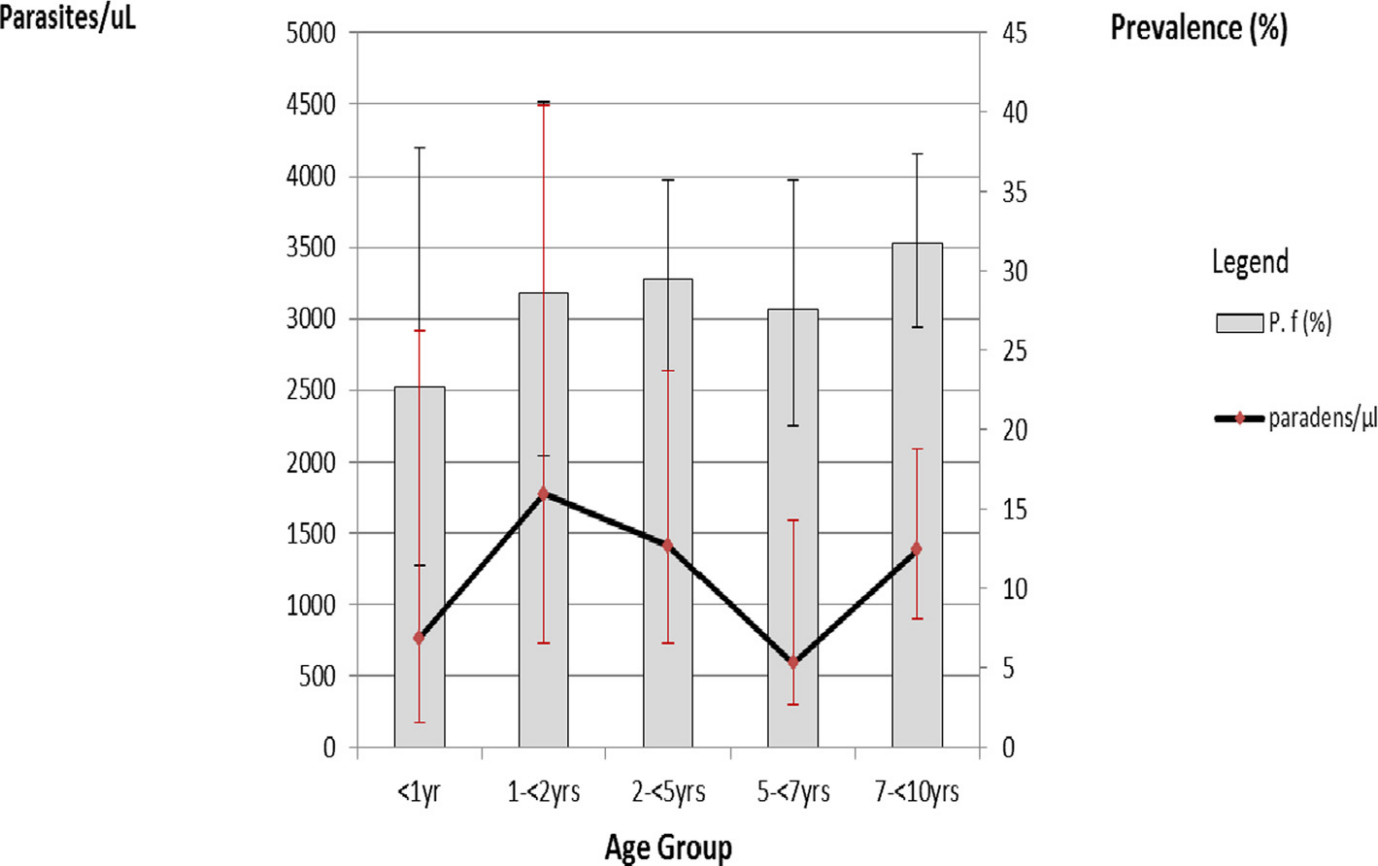
Mean parasite density and prevalence of infection by age group in children under 10 years of age in Nchelenge, Zambia (2013).

The prevalence of malaria infection was 30.2% (236/782), the large majority (98.0%, 231/236) *Plasmodium falciparum* and the remaining ones *Plasmodium malariae* (Table 2). No mixed infection was found. *Plasmodium falciparum* prevalence was the lowest in the <1 year old children while it varied between 22.7% (1 years old) and 31.8% (7–10 years old) in the other age groups (Table 2 and Figure 1).

**Table 2:** Malariometric indicators by age in children under 10 years from Nchelenge District, Zambia(N)

**Variable**	**Overall (782)**	**<12 m (44)**	**12-23 m (70)**	**24-59 m (234)**	**5- < 7 yrs (138)**	**7- < 10 yrs (296)**	**p**
*P. falciparum%* (n)	29.5 (231)	22.7 (10)	28.6 (20)	29.5 (69)	27.6 (38)	31.8 (94)	0.5
*P. malariae%* (n)	0.7 (5)	0.0	2.9 (2)	0.4 (1)	0.0	0.7 (2)	0.4
Gametocytes Pf% (n)	1.5 (12)	0.0	1.4 (1)	0.9 (2)	1.5 (2)	2.4 (7)	0.4
Mean Parasite density par/μl (95% CI)	1,250	765	1770	1410	590	1,380	0.7
	(944-1740)	(179-2920)	(726-4490)	(734-2640)	(299-1590)	(901-2090)	
Median haemoglobin, g/dl (IQR)	10.7	11.0	10.6	10.7	10.7	10.6	0.4
	(10.6-10.8)	(10.6-11.4)	(10.2-11.1)	(10.5-11.0)	(10.4-11.0)	(10.4-10.9)	
Anaemia (Hb < 11 g/dl)% (n)	51.2 (398)	47.7 (21)	55.7 (39)	47.9 (112)	49.3 (67)	54.1 (159)	0.4

m = months, yrs = years.

p-value used a t-test to compare proportions.

The geometric mean parasite density for *P. falciparum* was 1,250 parasites/μl (95% CI, 944–1,740) and varied with age, with the highest value in the 1–2 years old group, 1,770 parasites/μl (95% CI, 726–4,490), and the lowest, 590 parasites/μl (95% CI, 299–1,590) in the 5–7 years old group (p = 0.7) (Figure 1). Gametocytes were found in 12 (1.5%, 95% CI, 0.8-2.7) children, all of them *P. falciparum,* and were more frequent in the older age group (Table 2). No gametocytes were found in infected infants (<1 year old).

Mean Hb was 10.7 g/dl (95% CI, 10.6-10.8), ranging between 1.2 g/dl and 15.4 g/dl. About half (51.2% (398/782), 95% CI, 47.6-54.7%) of the children were anaemic (Hb < 11 g/dl) while 7.4% (58/782), (95% CI, 5.6-9.5) of them were severely anaemic (Hb < 8 g/dl). Mean Hb and anaemia did not vary with age (p = 0.7). Malaria infection was significantly associated with anaemia (AOR = 1.64, 95% CI, 1.20-2.24, p < 0.001) and such risk tended to be higher in children with the highest parasite densities (1.30, 95% CI, 0.07-22.74, p = 0. 86) (Table 3).

**Table 3:** Predictors for anaemia and malaria in children under 10 years from Nchelenge District, 2012, Zambia

***Risk factors for anaemia***	**Class**	**Unadjusted OR (95% CI)**	**Adjusted OR (95% CI)**	**P-value**
Malaria	+	2.11(1.56-2.85)	1.64 (1.20-2.24)^†^	<0.0001
	--	1.00	1.00	
Age-group	> 12 m	0.80 (0.42-1.53	1.22 (0.03-44.32)	0.81
	12-23 m	0.10 (0.57-1.65)	3.45 (0.50-23.94)	0.40
	24-59 m	0.80 (0.56-1.14)	1.83 (0.58-5.79)	0.91
	5- < 7 yrs	0.80 (0.54-1.19)	1.93 (0.38-0.89)	0.87
	7- < 10 yrs	1.00	1.00	-
Gender	Males	1.11 (0.83-1.49)	0.52 (0.19-1.40)	
	Females	1.00	1.00	0.46
Parasite density	>2000	1.20 (0.68-2.10)	1.30 (0.07-22.74)	0.86
	≤2000	1.00	1.00	-
***Risk factors for malaria infection***				
Age-group	> 12 m	1.00	1.00	-
	12-23 m	1.36 (0.57-3.26)	1.13 (0.17-7.58)	0.90
	24-59 m	1.42 (0.67-3.04)	4.23 (0.90-19.98)	0.06
	5- < 7 yrs	1.29 (0.58-2.87)	6.00 (1.22-29.51)	0.03
	7- < 10 yrs	1.58 (0.75-3.34)	2.94 (0.62-13.86)	0.17
Female	Males	1.00	1.00	
	Females	1.13 (0.82-1.54)	1.33 (0.73-2.42)^‡^	0.36
Mother education	Primary	1.00	1.00	
	Secondary	0.89 (0.51-1.67)	1.29 (0.53-3.13)^‡^	0.59
	Tertiary	0.83 (0.17-4.17)	0	0.97
Indoor residual spraying	Yes	1.00	1.00	
	No	0.80 (0.49-1.29)	0.81 (0.38-1.75)^‡^	0.59
Bed net use	Yes	1.00		
	No	1.48 (0.67-3.26)	1.70 (0.73-3.95)^‡^	0.22

^†^adjusted for age, gender, and parasite density. ^‡^adjusted for indoor residual spraying, age, gender and mother education. Age group was adjusted for bed net use and indoor residual spraying.

The risk of malaria infection was neither different in bed net users (AOR = 0.59, 95% CI, 0.31-3.95, p = 0.22) nor in households sprayed the previous year (OR = 1.23, 95% CI, 0.77-2.01, p = 0.36). The age specific odds show that children 3- < 5 years and those from 5- > 7 years who did not use bed nets the previous night and are in homes not sprayed are more likely to have a malaria infection than those who use bed nets and have their homes sprayed (OR = 6.00, 95% CI, 1.22-29.51, p = 0.03; OR = 4.23, 95% CI, 0.90-19.98, p = 0.06) (Table 3).

## Discussion

This malaria indicator survey, using the World Health Organization (WHO) survey tools, provides a recent estimation of the malaria burden in Nchelenge district. The prevalence of both malaria infection and anaemia was similar to those found in national surveys for Luapula province [13], a result that contrasts with the overall declining trend of malaria in Zambia [14]. Prior national surveys have reported a higher resurgence of malaria in Luapula province than other regions [5]. This could be attributed to seasonality of malaria.

It is important to notice that Nchelenge borders the Democratic Republic of Congo (DRC), with substantial population movements across the borders, and is difficult to reach due to poor terrain, possibly explaining the low coverage of control interventions. Indeed, only a third of the children included in the survey stated to have a bed net and even less used it the previous night. As IRS, only a small proportion of the sampled houses had received this intervention so that any impact on the local vector population is unlikely. It is unknown what the coverage of control interventions may be on the other side of the border but probably not higher than that in Nchelenge, more likely lower. In these conditions, the prevalence of malaria and anaemia found by this survey are not surprising as they reflect the extremely low coverage of ITN and IRS. However, even in case of a better coverage, prevalence may still have been high as the important population movements between Zambia and DRC would maintain the local human reservoir and hence malaria transmission. A significant reduction in the malaria burden in this region may be achieved only through a close collaboration between the national control programmes of both countries. Other countries sharing borders like South Africa, Mozambique and Swaziland in a programme called the Lubombo Spatial Development Initiative (LSDI) have achieved spectacular successes in reducing cross-border malaria burden [15].

The mean parasite density in the children included in this survey, most of them asymptomatic, was relatively high, particularly in the 1–2 years old age group, confirming Nchelenge as an area of intense malaria transmission. Prevalence of malaria would probably have been higher if molecular methods to detect infection had been used. Following continuous exposure to malaria infection, children develop partial immunity and are able to tolerate relatively high parasite densities. Access to treatment seems high as more than 60% of children’s parents stated they had taken an antimalarial treatment the month prior to the survey. However, information on the quality and the source of treatment was not collected during the survey so that it is impossible to know if this was of adequate quality and dosage. The apparently frequent administration of antimalarial treatment is just an indicator of the importance of malaria in this setting.

The prevalence of gametocytes was relatively low though probably a higher number of children with gametocytaemia could have been identified by using molecular methods. Detecting gametocytes in thick blood smears, particularly when present at low densities, is a challenge even for experienced microscopists [16]. Indeed, detecting of *P falciparum* gametocytes by microscopy can miss most of the gametocyte carriers [17-19] as they are present at an extremely low density though they can still be infectious to mosquitoes [20-22].

This was the weakness of this study in which gametocytes were examined on thick smears.

Coverage by bed nets and IRS was low and this may explain the higher prevalence of malaria infection. However, recent data seem to indicate a problem of emerging resistance to pyrethroids and DDT among the vector population in Nchelenge [23]. Neighbouring countries, such as Mozambique, have found similar patterns of resistance to pyrethroids [24]. If this is confirmed, increasing coverage of both insecticide-treated bed nets and IRS would have a lower than expected impact as ITN would just act as mechanical barriers.

The logistic multivariable analyses found that children did not use bed nets and are in households not sprayed have a higher risk of malaria infection in the age 2 to 7 years. This is the age group that is just weaned from the mother and would not be under a bed net nor cover themselves from mosquito bites. This study also confirms that malaria affects haemoglobin levels in communities where children bear the brunt of the malaria burden [25,26] and high levels of anaemia are seen as in other parts of Africa as Kenya and Tanzania [27,28]. Further, children with malaria infection have a higher risk of anaemia, having higher anaemia levels than those without malaria affecting children in many ways including poor performance [29].

The limitations of this work is that it was carried out in relatively smaller or narrow geographical focus. This could over- or underestimate the bed net or IRS coverage and indeed malaria prevalence. Since malaria presents with some seasonality, the study was done at the end of the rainy season but there could be differences in the dry season. Sociodemographic data were collected via self-reporting and, therefore, self-report, recall bias, and social desirability bias cannot be ruled out. Cognisant of this potential bias, the study interviewers made efforts to enhance truthful reporting. The nature of the cross-sectional survey does not permit causal inference of the observed association. In conclusion, even though overall figures indicate that malaria is declining in Zambia, there are still pockets of high endemicity such as the Nchelenge district. This district is on the shores of Lake Mweru and neighbouring DR Congo. Malaria will be controlled only by concerted efforts across borders aiming at increasing coverage of preventive intervention and prompt access to diagnosis and antimalarial treatment.

## Competing interests

The authors declare that they have no competing interests.

## Authors’ contributions

All authors contributed to the design of the study and assisted with data interpretation. MN and PM coordinated the study and supervised the enrolment, data collection and entry. PM did the laboratory analysis and study participants enrolment. JPVG, MM and UDA participated in analysis of data. All authors participated in the preparation of the manuscript and approved the final version. All read and approved the final manuscript.
